# Optimization of Vacuum Microwave-Mediated Extraction of Syringoside and Oleuropein from Twigs of *Syringa oblata*

**DOI:** 10.1155/2018/6179013

**Published:** 2018-09-05

**Authors:** Xiangping Liu, Xuemin Jing, Guoliang Li

**Affiliations:** College of Animal Science and Veterinary Medicine, Heilongjiang Bayi Agricultural University, Daqing 163319, China

## Abstract

A vacuum microwave-mediated method was used to extract syringoside and oleuropein from *Syringa oblata* twigs. The optimal extraction conditions were an ethanol volume fraction of 40%, a liquid-solid ratio of 17 mL/g, 1 h of soaking time, −0.08 MPa of vacuum, a microwave irradiation power of 524 W, and a microwave irradiation time of 8 min. Under optimal parameters, the maximum yields of syringoside (5.92 ± 0.24 mg/g) and oleuropein (4.02 ± 0.18 mg/g) were obtained. The proposed method is more efficient than conventional methods for extracting syringoside and oleuropein from *Syringa oblata*. Moreover, less energy and time were required. The results implied that vacuum microwave-mediated extraction is a suitable method for the extraction of thermosensitive glycosides such as syringoside and oleuropein.

## 1. Introduction

Recently, many studies have been done on the utilization of microwave-assisted extraction (MAE) to obtain biologically active analytes such as glycosides [1–4], phenolcarboxylic acids [5, 6], procyanidins [7], stilbenes [8], coumarins [9], alkaloid [10], pectin [11], and essential oil [12]. MAE is a process by which microwave energy is used to heat the sample and the solvent, leading to fast movement of the target component molecules from the sample into the extracting solvent. Extraction using microwaves has advantages over traditional extraction techniques, including greater efficiency, less solvent consumption, and reduced extraction times. However, the reaction temperature of MAE is typically near the boiling point of the extraction solvent at atmospheric pressure, which causes the degradation or oxidation of some thermolabile and oxygen-sensitive secondary metabolites in traditional MAE systems [13, 14]. Vacuum microwave-mediated extraction (VMME) is a good alternative technique to prevent degradation of thermal-sensitive and oxygen-sensitive compounds at high extraction temperatures in open MAE [15–19]. Additionally, the exclusion of air from the extraction system can increase the extraction yield as well [17, 20, 21].

*Syringa* is a genus of the Oleaceae family and is widely cultivated in many warm-temperate countries [22]. China is a rich natural resource center for *Syringa* [23]. Of the *Syringa* species, *Syringa oblata* is widely grown in regions of northern China. In addition to being widely grown for landscape greenery, *S. oblata* has been used as folk medicine for treating various diseases for many centuries [24].

**Figure 1:**
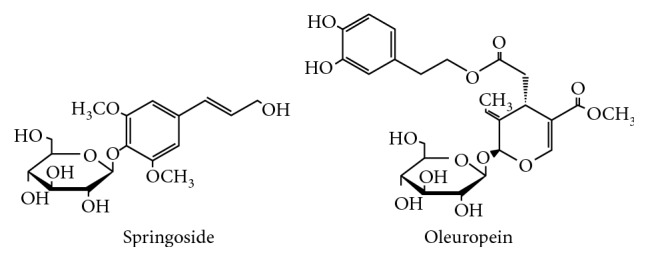
The chemical structures of syringoside and oleuropein.

Syringoside and oleuropein are the important active ingredients of *S. oblata* [25, 26]. Syringoside (Figure 1) is commonly regarded as a significant phenylpropanoid glycoside [27]. Pharmacology studies have demonstrated that syringoside has anti-inflammatory and pain-suppressing activities [28], immune regulation activity [29], neuroprotective effects [30], and activity in reducing injury from ultraviolet radiation [31], and it promotes insulin secretion [32, 33]. Oleuropein is a secoiridoid glucoside [27] (Figure 1). Oleuropein possesses significant antioxidant activity and has various pharmacological effects, including anti-inflammatory, antitumor, antibacterial, antiviral, antiarteriosclerosis, hypolipidemic, hypoglycemic, and hepatoprotective activities [34, 35].

Heat reflux extraction (HRE) has been traditionally used for the extraction of glycosides from plants. However, degradation by hydrolysis isomerization, condensation, or oxidation during HRE contributes to the loss of glycosides. These problems make this technique inefficient and lead to low recovery of these compounds.

In this study, an effective and environmentally friendly vacuum microwave-mediated extraction (VMME) method was used to extract syringoside and oleuropein from twigs of *S. oblata.* VMME parameters were optimized. Moreover, the extraction efficiency of the VMME technique was compared with the traditional HRE technique for the extraction of syringoside and oleuropein from *S. oblata*.

## 2. Experimental

### 2.1. Materials and Reagents

Twigs were manually picked from similar nearly 2.5 m, 10-year-old cultivated *S. oblata* trees in September 2017 in the Daqing suburbs, Heilongjiang Province, China. The twigs were dried at room temperature to a constant weight, and the cut pieces were ground using a laboratory mill and sieved (50-mesh screen). Powdered materials were stored in large brown bottles until use. Reference syringoside and oleuropein were obtained from Sigma-Aldrich (Shanghai, China).

### 2.2. VMME Apparatus

A modified microwave-assisted extraction unit (WP700, Galanz Enterprise Co, Ltd., Guangdong, China) was utilized for the experiment. A water condenser coated with PTFE was connected to the top of microwave [36]. Between the condenser and the flask, the extraction system was connected to a vacuum pump (SHB-III, Shanghai Yuezhong Equipment Co., Ltd, Shanghai, China) to generate a vacuum. The extraction system provided an inner microwave volume of 21.5 × 35 × 33 cm^3^. It operates at a frequency of 2540 Hz and can continuously transmit microwave energy to the reactor.

### 2.3. VMME Procedure

The powdered samples (1.0 g) were placed in 100 mL flasks. Then, the extraction agent was added depending on the experimental design. The flask was linked to the condenser through a hole on the microwave oven. The air in the flask was evacuated by a vacuum pump until the required vacuum was acquired. Before extraction, the material was soaked in the solvent for specific times to improve microwave absorption. VMME was conducted under various experimental conditions. The influences of ethanol volume fraction, soaking time, liquid-solid ratio, degree of vacuum, microwave irradiation power, and time on the extraction yields of syringoside and oleuropein were systematically investigated. After each extraction, the supernatant was filtered through a 0.45 *μ*m membrane and then applied to RP-HPLC.

### 2.4. Optimization of VMME by RSM

To investigate the optimal reaction parameters of the VMME, Design Expert (Version 8.0.6) was used to optimize the operating conditions by RSM. An experiment model based on a response pattern was established through the Box–Behnken design (BBD) in Design Expert. Considering the results of preliminary individual factor experiments, microwave irradiation time, liquid-solid ratio, and microwave irradiation power were chosen as key variables and assigned as *X*_1_, *X*_2_, and *X*_3_, respectively. The range of the factors were 4–8 min for the microwave irradiation time, 10–20 mL/g for the liquid-solid ratio, and 230–540 W for the microwave irradiation power, as shown in Table 1. The experiment model designed through BBD included 17 experiments. The extractions were performed in a random order. The three independent variables were related to two dependent variables, the yield of syringoside (*Y*_1_) and oleuropein (*Y*_2_). The complete quadratic equation is as follows:

(1) $$\begin{array}{c} Y=\beta_{0}+\overset{3}{\underset{i=1}{\sum }}\beta_{i}X_{i}+\overset{3}{\underset{i=1}{\sum }}\beta_{ii}X_{i}^{2}+\overset{2}{\underset{i=1}{\sum }}\overset{3}{\underset{j=i+1}{\sum }}\beta_{ij}X_{i}Y_{j}, \end{array}$$

where *Y* represents the estimated response; *β*_0_ is an intercept; *β*_*i*_, *β*_*ii*_, and *β*_*ij*_ represent the regression coefficients for linear, quadratic, and interactive terms, respectively; and *X*_*i*_ and *X*_*j*_ represent the independent variables.

### 2.5. HPLC Analysis

HPLC analysis of syringoside and oleuropein was conducted through a Waters Millennium32 system. The Waters HPLC system consisted of a 717 automatic sampler, a 717 automatic column temperature control box, a 1525 pump, and a 2487 UV detector. Chromatographic separation was performed on a Zorbax XDB-C18 column (4.6 mm × 250 mm, 5 *μ*m, Agilent). Methanol with 0.2% phosphoric acid (33 : 67, v/v) was employed as the mobile phase. The wavelength applied to syringoside and oleuropein for the diode detector was 232 nm. Flow rate was 1.0 mL/min, the injection volume was 10 *μ*L, and the column temperature was maintained at 25°C. Syringoside and oleuropein were quantitatively analyzed by a standard analytical method. Calibration curves for syringoside and oleuropein were established in a concentration range of 0.1–0.5 mg/mL. The linear regression equations for syringoside and oleuropein were *Y*_syringoside_ = 18914*X* + 17067 (*r*^2^ = 0.9998, *n* = 8) and *Y*_oleuropein_ = 15677*X* − 72912 (*r*^2^ = 0.9993, *n* = 8), respectively.

## 3. Results and Discussion

### 3.1. Individual Factor Tests of VMME Parameters

The individual factor experiments were conducted to optimize the following parameters: ethanol volume fraction, soaking time, vacuum, liquid-solid ratio, microwave irradiation power, and microwave irradiation time.

#### 3.1.1. Effect of Ethanol Volume Fraction

**Figure 2:**
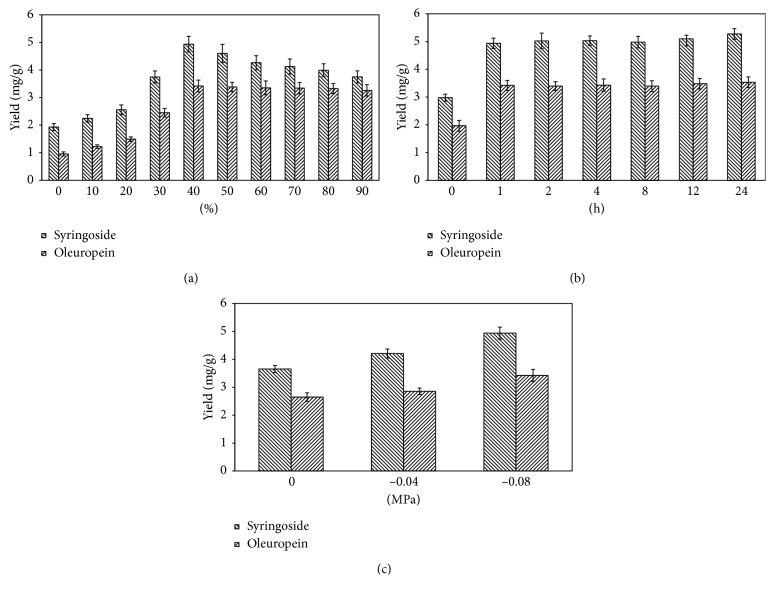
Effects of the ethanol volume fraction (a), soaking time (b), and level of vacuum (c) on the extraction yields of the target analytes. Error bars indicate standard deviation (*n*=3).

Ethanol has low toxicity and is widely used in commercial processes [37]. The extractions were conducted in various ethanol volume fractions from 0 to 80%. The remaining variables were vacuum −0.08 MPa, soaking time 1 h, liquid-solid ratio 20 mL/g, microwave irradiation time 6 min, and microwave irradiation power 385 W.  Figure 2(a) shows the significant effect of ethanol volume on extraction yields of syringoside and oleuropein. The extraction yields of syringoside and oleuropein increased with an increasing ethanol volume fraction from 0 to 40%. With further increase in the volume of ethanol, the yield of syringoside and oleuropein decreased gradually. This indicates that using sufficient ethanol enhanced the extraction efficiency for the two compounds, but too much ethanol was disadvantageous for the extraction of syringoside and oleuropein. An appropriate volume of ethanol also contributed to decreasing the viscosity of the extraction solvent and enhancing the permeability of the substrate, which made the target components more easily extracted. However, because syringoside and oleuropein are glycosides, they have similar properties as sugars, so excessive ethanol in the extraction solvent decreased the solubility of syringoside and oleuropein. Based on these data, a 40% ethanol volume fraction was selected for subsequent RSM experiments.

#### 3.1.2. Effect of Soaking Time

To select a proper soaking time, the dry herb powder was soaked in the ethanol solution for 1, 2, 4, 8, 12, and 24 h before VMME. These experiments were performed in a 40% (volume concentration) ethanol solution, and the results are shown in Figure 2(b). The extraction yield of syringoside and oleuropein was highly influenced by soaking time. To extract target analytes from plant cells, cell contents must be accessible to the solvent. Syringoside and oleuropein yields were increased by soaking because soaking improved penetration of the solvent into the cells, allowing increased solubilization of the target analytes. A 1 h soaking time greatly enhanced the yields of syringoside and oleuropein; however, this improvement was limited with further extension of the soaking time. Therefore, 1 h was chosen as the optimal soaking time.

#### 3.1.3. Effect of the Degree of Vacuum

To study the effect of the degree of vacuum on extraction yields of syringoside and oleuropein, several experiments were conducted at three different levels of vacuum with other extraction parameters set to 40% ethanol as the extraction solvent, 1 h as the soaking time, 20 mL/g as the liquid-solid ratio, 6 min as the microwave irradiation time, and 385 W as the microwave irradiation power. The results are shown in Figure 2(c). A higher degree of vacuum, −0.08 MPa, was appropriate for extraction of syringoside and oleuropein in MAE, and a weaker vacuum, 0.04 MPa, was not helpful for the extraction of these compounds. The reason for the increased yields might be due to the lower solvent boiling point caused by the higher degree of vacuum, which reduced the degradation of syringoside and oleuropein.

#### 3.1.4. Effect of the Liquid-Solid Ratio

**Figure 3:**
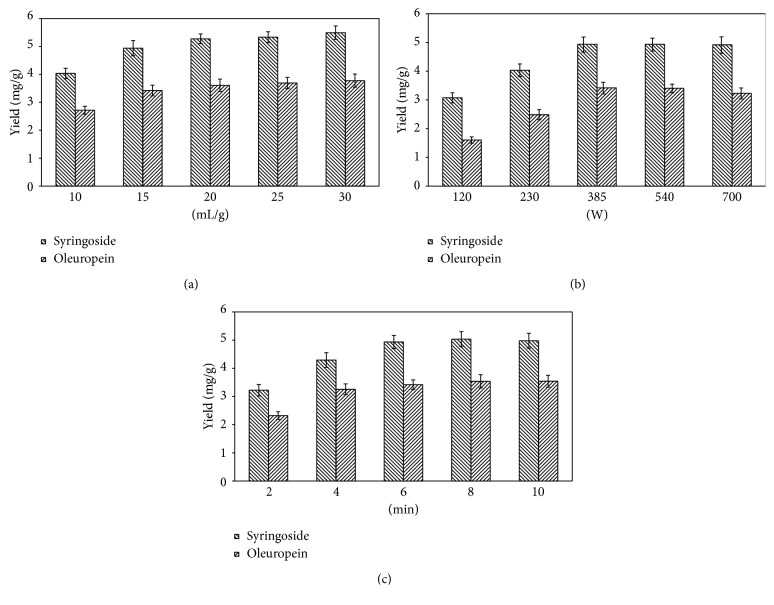
Effects of the liquid-solid ratio (a), microwave irradiation power (b), and microwave irradiation time (c) on the extraction yields of the target analytes. Error bars indicate standard deviation (*n*=3).

The liquid-solid ratio is a key variable that was also investigated to optimize extraction efficiency. A low solvent amount may result in insufficient extraction, whereas large volumes can lead to unnecessary cost and make the procedure difficult. Five different liquid-solid ratios (10, 15, 20, 25, and 30 mL/g) were tested. The remaining variables used for these experiments were degree of vacuum −0.08 MPa, ethanol volume fraction 40%, microwave power 230 W, microwave time 6 min, and soaking time 1 h.  Figure 3(a) shows that the extraction efficiencies of both compounds were enhanced with a liquid-solid ratio of up to 20 mL/g, but increasing the liquid-solid ratio further resulted in no significant additional enhancement of extraction yields. Therefore, 10–20 mL/g was selected as the proper liquid-solid ratio to save solvent and costs.

#### 3.1.5. Effect of Microwave Irradiation Power

To understand the influence of microwave power on VMME, different power levels were tested. The other variables used in these experiments were degree of vacuum −0.08 MPa, ethanol volume fraction 40%, liquid-solid ratio 20 mL/g, soaking time 1 h, and microwave irradiation time 6 min. As shown in Figure 3(b), the yields of both syringoside and oleuropein reached a peak at 385 W. When the microwave power was 540 W or higher, the yields of both compounds did not increase further. Thus, 230–540 W was selected for the following experiments to achieve optimal reaction parameters of the VMME.

#### 3.1.6. Effect of Microwave Irradiation Time

Extraction time is a crucial factor that must be studied to guarantee complete extraction of active compounds, regardless of the technique. The impact of different extraction times on the yields of syringoside and oleuropein was evaluated (Figure 3(c)) under the following conditions: degree of vacuum −0.08 MPa, ethanol volume fraction 40%, liquid-solid ratio 20 mL/g, microwave irradiation power 385 W, and soaking time 1 h. With an increase of irradiation time from 2 to 6 min, the extraction efficiency of syringoside improved. However, further increases in microwave irradiation times beyond 6 min did not improve the syringoside extraction efficiency. Additionally, the yield of oleuropein increased from 2 min to 4 min, and the extraction efficiency correlated with the microwave irradiation time. Based on these results, VMME was conducted with an extraction time of 4–8 min in subsequent experiments.

### 3.2. Optimization of the VMME Parameters Using BBD

RSM design was used to determine the optimal VMME parameters for extracting syringoside and oleuropein from *S. oblata*. According to the results of the individual factor experiments, three different factors, liquid-solid ratio, microwave irradiation power, and time, were chosen for following optimizing experiments using BBD. The optimal VMME conditions for vacuum, microwave irradiation power, and soaking time in the individual factor experiments were found to be −0.08 MPa, 120 W, and 1 h, respectively, and these values were used in the BBD experiments, which comprised 17 experiments (Table 1). Predicted data were obtained by RSM from mathematical models.

Variance analysis of the RSM was conducted to analyze the significance and suitability of the mathematical models (Table 2). Regression models with *P* values less than 0.001 indicated that syringoside and oleuropein yields predicted by the two models were adequate. Moreover, *P* values of “lack of fit” were 0.4263 and 0.2684 for syringoside and oleuropein, respectively, which showed that the “lack of fit” was not significantly correlated with pure error due to statistical noise. There was a significant correlation between the predicted and actual yield for syringoside and oleuropein. The two regression models for syringoside and oleuropein yields, with *R*^2^ values of 0.9885 and 0.9811, respectively, indicated that these models can explain 98.85% and 98.11% of the variation in the response. The following mathematical models were obtained:

(2) $$\begin{array}{c} Y_{\text{syringoside}}=-6.97+1.95X_{1}+1.05\times 10^{-2}X_{3}+1.42\times 10^{-3}X_{1}X_{3}-1.95\times 10^{-1}X_{1}^{2}-1.98\times 10^{-5}X_{3}^{2}, \end{array}$$

(3) $$\begin{array}{c} Y_{\text{oleuropein}}=0.85+1.77\times 10^{-1}X_{1}+7.99\times 10^{-3}X_{3}+1.62\times 10^{-3}X_{1}X_{3}-7.30\times 10^{-2}X_{1}^{2}-4.38\times 10^{-4}X_{2}^{2}-1.77\times 10^{-5}X_{3}. \end{array}$$

**Figure 4:**
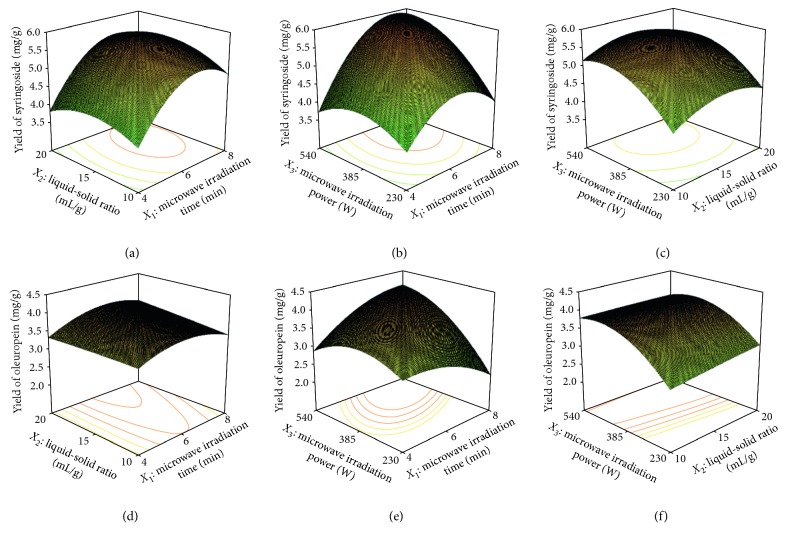
Response surface plots showing the effects of variables on yields of syringoside and oleuropein: (a) interaction between microwave irradiation time and liquid-solid ratio on the extraction yield of syringoside; (b) interaction between microwave irradiation time and microwave irradiation power on the extraction yield of syringoside; (c) interaction between liquid-solid ratio and microwave irradiation power on the extraction yield of syringoside; (d) interaction between microwave irradiation time and liquid-solid ratio on the extraction yield of oleuropein; (e) interaction between microwave irradiation time and microwave irradiation power on the extraction yield of oleuropein; (f) interaction between liquid-solid ratio and microwave irradiation power on the extraction yield of oleuropein.

To research the effects of the three parameters and their interactions on the yields of syringoside and oleuropein, 3D response surface graphs were drawn according to mathematical models (Equation (2) and Equation (3)). The effects of *X*_1_ and *X*_2_ on the extraction yields of two target compounds are shown in Figures 4(a) and 4(d), respectively, at a fixed *X*_3_ of 385 W. At the early stage of extraction, both compounds increased in the extraction yield with extraction time; however, this was followed by slightly decreased extraction yields with further increases in extraction time. These results demonstrated that appropriate microwave irradiation time is necessary for completely extracting the target compounds. Increases in the liquid-solid ratio have little effect on the extraction yield of oleuropein. However, for syringoside, the liquid-solid ratio had a positive linear correlation with the yield. A higher liquid to solid ratio is favorable for permeation of the solvent and discharge of target components due to the difference in the target compounds' concentration on either side of the cell membrane. Figures 4(b) and 4(e) illustrate the effect of *X*_1_ and *X*_3_ on the yields of syringoside and oleuropein with a fixed *X*_2_ (15 mL/g). Here, a similar trend for syringoside and oleuropein yields was observed. The maximal syringoside and oleuropein yields were obtained at higher microwave irradiation power and longer microwave irradiation time.  Figure 4(c) and 4(f) shows the interaction of *X*_2_ and *X*_3_ at a fixed *X*_1_ (6 min). Microwave irradiation power had a greater effect on the extraction yields of syringoside and oleuropein than the liquid-solid ratio.

The optimal parameters in the extraction of syringoside and oleuropein by VMME predicted by BBD analysis were as follows: microwave irradiation time, 8 min; liquid-solid ratio, 17 mL/g; and microwave irradiation power, 524 W. Using these optimal conditions, the predicted yields of syringoside and oleuropein were 6.0 mg/g and 4.1 mg/g, respectively.

### 3.3. Verification

Five extractions were conducted with the optimal conditions (microwave time, 8 min; liquid-solid ratio, 17 mL/g; and microwave power, 524 W) to evaluate the reliability of VMME for extracting syringoside and oleuropein. Using the optimal parameters described above, the actual experimental values of syringoside and oleuropein were 5.92 ± 0.24 mg/g and 4.02 ± 0.18 mg/g, respectively, which were consistent with the predicted values. Therefore, the optimal conditions obtained by the RSM method were reliable and reasonable.

### 3.4. Comparison of Different Extraction Procedures

A comparison among VMME, MAE, and HRE is shown in Table 3 for the yields of syringoside and oleuropein from *S. oblata* twig samples. The extraction yields of analytes in VMME were higher and required a shorter extraction time compared to MAE and HRE using optimal conditions. The superiority of VMME is mainly due to the low pressure and temperature accomplished by introducing vacuum pressure in MAE, which decreases the degradation of thermosensitive and oxidizable compounds. Compared with conventional HRE, VMME and MAE are faster, while obtaining higher extraction yields for the target analytes. The effect of the microwave is to diffuse the inner components, change the internal microscopic structure, and cause the swelling of cells or the breakdown of cell walls when the temperature rises, which enhances the mass transfer of the cell contents [38, 39]. The comparison of VMME to MAE showed that the vacuum was important for increasing the extraction yields. These results are consistent with studies on VMME of vitamin C, vitamin E, and polyphenolic compounds [15, 16]. Therefore, we conclude that the VMME procedure provides a rapid and effective approach for the extraction of thermosensitive glycosides from the plant or other materials.

## 4. Conclusions

An effective VMME method was presented for extracting syringoside and oleuropein from *S. oblata* twigs. The optimal conditions for VMME were explored using an RSM method. With the optimal conditions, maximum extraction yields of the two glycosides were obtained. Relative to MAE and HRE, the VMME approach had significantly higher extraction yield. VMME exhibited good extraction efficiencies for extracting syringoside and oleuropein. This method avoids the high temperatures of conventional extraction methods, which may also prove useful as a promising extraction method for other glycosides.

## Figures and Tables

**Table 1:** Experimental data and the observed response value with different combinations of microwave irradiation time (*X*_1_), liquid-solid ratio (*X*_2_), and microwave irradiation power (*X*_3_) used in the Box–Behnken design.

Run number	Experimental design			Dependent variables			
	*X*_1_: microwave irradiation time (min)	*X*_2_: liquid-solid ratio (mL/g)	*X*_3_: microwave irradiation power (W)	Yield of syringoside (mg/g)		Yield of oleuropein (mg/g)	
				Predicted yield	Actual yield	Predicted yield	Actual yield
1	4	10	385	3.82	3.82	3.39	3.44
2	8	10	385	4.86	4.94	3.43	3.40
3	4	20	385	3.81	3.73	3.35	3.37
4	8	20	385	5.27	5.27	3.72	3.67
5	4	15	230	3.65	3.63	3.02	2.92
6	8	15	230	4.02	3.92	2.22	2.20
7	4	15	540	3.73	3.83	2.89	2.91
8	8	15	540	5.86	5.88	4.10	4.20
9	6	10	230	4.13	4.14	2.77	2.82
10	6	20	230	4.40	4.51	3.04	3.11
11	6	10	540	5.16	5.05	3.78	3.70
12	6	20	540	5.29	5.28	3.77	3.73
13	6	15	385	5.57	5.71	3.78	3.89
14	6	15	385	5.57	5.57	3.78	3.78
15	6	15	385	5.57	5.52	3.78	3.76
16	6	15	385	5.57	5.39	3.78	3.65
17	6	15	385	5.57	5.68	3.78	3.81

**Table 2:** Estimated regression coefficients for the quadratic polynomial model and ANOVA for the experimental results in the optimization of syringoside and oleuropein extractions^a^.

Regression coefficients	Sum of squares		Degree of freedom	Mean square		*F* value		Probability >*F*	
	Y_oleuropein_^b^	Y_syringoside_		Y_oleuropein_	Y_syringoside_	Y_oleuropein_	Y_syringoside_	Y_oleuropein_	Y_syringoside_
Model	10.331	3.873	9	1.148	0.430	66.57	40.48	<0.0001^*∗∗∗*^	<0.0001^*∗∗∗*^
*X*_1_^b^	3.125	0.083	1	3.125	0.083	181.24	7.79	<0.0001^*∗∗∗*^	0.0269
*X*_2_	0.085	0.034	1	0.085	0.034	4.95	3.18	0.0614	0.1178
*X*_3_	1.835	1.514	1	1.835	1.514	106.43	142.38	<0.0001^*∗∗∗*^	<0.0001^*∗∗∗*^
*X*_1_ *X*_2_	0.045	0.028	1	0.045	0.028	2.62	2.67	0.1494	0.1465
*X*_1_ *X*_3_	0.777	1.009	1	0.777	1.009	45.05	94.89	0.0003^*∗∗∗*^	<0.0001^*∗∗∗*^
*X*_2_ *X*_3_	0.005	0.018	1	0.005	0.018	0.30	1.66	0.6036	0.2383
*X*_1_^2^	2.569	0.359	1	2.569	0.359	148.96	33.75	<0.0001^*∗∗∗*^	0.0007^*∗∗∗*^
*X*_2_^2^	0.525	0.001	1	0.525	0.001	30.47	0.05	0.0009	0.8336
*X*_3_^2^	0.956	0.760	1	0.956	0.760	55.45	71.48	0.0001^*∗∗∗*^	<0.0001^*∗∗∗*^
Lack of fit	0.056	0.044	3	0.019	0.015	1.17	1.92	0.4263	0.2684

Credibility analysis of the regression equations	Index mark	Standard deviation	Mean	CV %	Press	R^2^	Adjust R^2^	Predicted R^2^	Adequacy precision

	Y_oleuropein_	0.13	4.82	2.73	1.00	0.9885	0.9736	0.9042	21.92
	Y_syringoside_	0.10	3.43	3.00	0.75	0.9811	0.9569	0.8100	23.70

^a^The results were obtained with Design Expert 8.0.6 software; ^b^*X*_1_ is microwave irradiation time (min); *X*_2_ is liquid-solid ratio (mL/g); *X*_3_ is microwave irradiation power (W); *Y*_oleuropein_ and *Y*_syringoside_ are the yield of oleuropein and syringoside (mg/g); ^*∗*^*p* < 0.05, significant;^ *∗∗*^*p* < 0.01, highly significant;^*∗∗∗*^*p* < 0.001, extremely significant.

**Table 3:** Comparison of extraction by different methods.

Method	Solvent	Soaking time (h)	Heating time consumption (min)	Yield (mean ± SD) (mg/g)	
				Syringoside	Oleuropein
VMME	40% volume fraction ethanol	1	6	5.92 ± 0.24	4.02 ± 0.18
MAE	40% volume fraction ethanol	1	6	3.95 ± 0.14	3.02 ± 0.09
HRE	40% volume fraction ethanol	1	60	2.52 ± 0.08	2.31 ± 0.08

VMME, vacuum microwave-mediated extraction; MAE, microwave-assisted extraction; HRE, hot reflux extraction.

## Data Availability

The data used to support the findings of this study are available from the corresponding author upon request.
